# Mental health practitioners’ experiences providing mental healthcare services to students in a higher education institution: A qualitative study

**DOI:** 10.4102/sajpsychiatry.v32i0.2649

**Published:** 2026-07-31

**Authors:** Simangele L. Linda, Nompumelelo Ndlovu, Annie Temane

**Affiliations:** 1 Department of Nursing, Faculty of Health Sciences, University of Johannesburg, Johannesburg, South Africa

**Keywords:** higher education institutions, mental health practitioners, mental healthcare services, qualitative research, student mental health, student support services

## Abstract

**Background:**

Mental healthcare service provision is challenging and becomes more complex when users are students. Students are more vulnerable to experiencing mental health challenges. Therefore, higher education institutions (HEIs) must offer effective mental healthcare services to meet the mental health needs of students.

**Aim:**

To understand the experiences of mental health practitioners (MHPs) who work for a HEI providing mental healthcare services.

**Setting:**

The study was conducted in South Africa within the context of higher education.

**Methods:**

A qualitative, exploratory, descriptive and contextual research design, incorporating the Appreciative Inquiry 5D Model, was used. This method allowed for the exploration and description of the experiences of MHPs working in a higher education setting who are tasked with providing mental healthcare services to students.

**Results:**

Five themes emerged from the analysis of data: (1) mental healthcare services are viewed as a source of holistic support, (2) mental healthcare provision is experienced as fulfilling and challenging, (3) mental healthcare services should be student-centred and engaging, (4) services should be available, accessible and responsive to address students’ needs, (5) mental healthcare services should integrate monitoring and evaluation strategies.

**Conclusion:**

Resource challenges and the rising demand for mental healthcare services by students remain an enormous burden on the already-burdened healthcare system. Higher education institutions must commit to funding these services, and then MHPs will be able to provide effective mental healthcare services to the students.

**Contribution:**

Strategies to guide the facilitation of students’ mental health were developed.

## Introduction

University students are the human capital for any nation.^1^ Obtaining a university qualification is viewed as the key to success and a bright future, boosting the country’s economy. However, mental health challenges among students are an area of concern,^2,3^ with research showing a decline in students’ mental health. It is estimated that one in five students has a diagnosable mental illness,^4^ and not less than 60% met one of the two diagnosable mental health criteria.^5^ The prevalent mental health issues among students include stress, anxiety, depression, post-traumatic stress disorder, eating disorders and suicidal ideation.^5,6^

Mental health practitioners (MHPs) are on the frontlines of assisting clients with mental health challenges in the general population.^7^ Mental health practitioners consist of psychiatrists, psychologists, social workers, nurses, counsellors and therapists.^7^ In the context of higher education institution (HEI), MHPs are responsible for providing mental healthcare services to students. They support students in navigating and coping with academic, personal and mental health challenges^8^; therefore, mental healthcare services are crucial in these settings.^9^ Unmet mental health needs of students pose challenges; hence, establishing mental healthcare services in HEIs is important.^5,7,9^ In South Africa, the *#FeesMustFall and #RhodesMustFall* movements exposed many challenges, including social inequalities and exclusions, which led to students experiencing mental health challenges.^10^ A study based in the United Kingdom supported this finding that most students experienced mental health challenges before enrolling in the university.^11^ Post-coronavirus disease 2019 (COVID-19), studies report rising demands for mental healthcare services in HEIs.^7,8,12^ The rise in mental health challenges places severe strain on academic and therapeutic staff.^2,13^ Mental health practitioners find it challenging to meet students’ mental health needs and manage their own mental health. This scourge amid resource shortage creates serious barriers to mental healthcare service provision and access by students of HEIs.^7^ Poor resource allocation for the provision of mental healthcare services in HEIs results in negative consequences for students, the institution and MHPs’ overall function. Previous studies have reported that students complain about long waiting periods to access mental healthcare services and to consult with MHPs; some studies have highlighted a high dropout rate among students due to unmet mental health needs, poor coping skills and, more seriously, the suicide ideation rate that remains high.^14^ Mental health practitioners are tasked with providing students with mental health support, with fewer resources. To date, no studies have explored the experiences of MHPs working at HEIs in South Africa. However, few studies focused on the experiences of academic staff in supporting students with mental health challenges. Results from those studies showed that academic staff felt overwhelmed, incapacitated and unsure of how to help the students.^11,15^ This study aimed to explore the experiences of MHPs working at HEIs in providing mental healthcare services to students in the context of poor resource allocation, effects of COVID-19 and financial constraints. The research question posed was: ‘*What are the experiences of MHPs when providing mental healthcare services?*’ The study concludes with suggestions and recommendations based on shared experiences among MHPs.

## Research methods and design

A qualitative, exploratory, descriptive and contextual research design incorporating the Appreciative Inquiry (AI) 5D Model was used. Qualitative research is an organised, collaborative and holistic scholarly approach that explores and describes human life experiences.^16,17^ Descriptive research accurately portrays specific characteristics of phenomena,^16^ while exploratory designs are used to determine what is happening, generate new insights and examine phenomena from a fresh perspective.^18^ A naïve sketch was used as an initial exploratory method to obtain a broad overview of participants’ experiences. Contextual research involves observing and engaging with participants within their natural settings.^19^ Appreciative Inquiry, as a research method, builds on participants’ positive experiences to stimulate change and serves as a motivational organisational change strategy to improve the quality and safety of healthcare.^20,21^

### Setting

The study was conducted in an HEI in the Gauteng province of South Africa. Data were collected in the researcher’s consultation room at a mental healthcare clinic.

### Study population and sampling

The study’s population consisted of MHPs employed by the HEI to provide mental health care services to students. Mental health practitioners are psychologists, psychology interns and social workers employed at the HEI. A total of 12 MHPs were recruited using purposive and snowball sampling. The researcher approached MHPs in person to deliver the research letters, and only those who expressed interest in participating were formally invited. Individual meetings were held to provide details about the research. Participants who were willing were asked to sign two written consent forms confirming their participation and permission for audio recording.

### Inclusion and exclusion criteria

Participants were MHPs or interns employed full-time by the HEI. The inclusion criteria were that all participants must have clinical experience as counsellors, therapists or psychiatrists providing mental health services to students within an HEI setting. Mental health practitioners not employed full-time, including professionals working on a part-time or contractual basis were not included. Also, MHPs on probation and those employed as academics were excluded. Table 1 presents a summary of participants’ demographics, organised by the following headings: gender, age and years of employment with the institution.

**TABLE 1:** A summary of the demographics of mental health practitioners.

Participant no.	Gender	Age (years)	Years employed by the HEI
MHP1	Female	32	5
MHP2	Female	33	7
MHP3	Female	49	4
MHP4	Female	54	18
MHP5	Male	44	14
MHP6	Female	59	21
MHP7	Male	27	1
MHP8	Female	26	1
MHP9	Female	25	1
MHP10	Female	25	1
MHP11	Non-binary	38	5
MHP12	Female	36	1

*Source*: Adapted from Simangele LL. Strategies to facilitate the mental health of students in a higher education institution [homepage on the Internet]. University of Johannesburg; 2025 [cited 2025 Nov 13]. Available from: https://ujcontent.uj.ac.za/esploro/outputs/doctoral/Strategies-to-facilitate-the-mental-health/9956306507691#file-0^22^

HEI, higher education institution; MHP, mental health practitioner; no., number.

### Data collection

Data were collected at the MHP’s offices over 11 months (April 2022–February 2023) using in-depth individual phenomenological interviews and naïve sketches. Naïve sketches are artworks created by self-taught or amateur artists; they are often seen as illustrating realism and expressing the artist’s feelings without applying academic training rules.^23^ The naïve sketches were voluntary and not requested from all participants. The pilot study was conducted with one participant to assess the suitability of the research technique and identify any methodological shortcomings. The findings were incorporated into the results. The pilot was successful, as the participant understood the questions and the researcher demonstrated confidence in conducting the interview. The interview was transcribed and reviewed by two supervisors to confirm the appropriateness of the questions before proceeding with further interviews. Data saturation was reached with the seventh participant, but the researcher conducted an additional five interviews for confirmation. Field notes were collected during the interviews and submitted for data analysis. The researcher used the AI 5D Model to conduct the interviews. The following were the research questions:

- *Define: What, in your view, are mental healthcare services?*
- *Discover: Tell me about your experiences when providing mental healthcare services*.
- *Dream: Imagine the best mental healthcare services*.
- *Design: What, in your view, are ideal mental healthcare services?*
- *Deliver: How can you ensure that the mental healthcare services are meeting students’ mental health needs?*

### Data analysis

Data analysis was conducted using Giorgi’s data analysis method.^24^ The raw collected data were first transcribed verbatim, and the transcriptions were read to identify meaning and then develop themes and sub-themes. An independent coder assisted in developing the themes, which were then submitted to the supervisors and finalised through consensus meetings between the researcher and the supervisors.

### Ethical considerations

Ethical approval to conduct this study was obtained from the University of Johannesburg Research Ethics Committee (Ref. No. REC-1307-2021). The researcher obtained written consent from all willing participants before conducting the interviews. No participants were coerced into participating, and they were informed that they could withdraw at any time within the first 10 min of the interview.

## Results

Five themes and eight sub-themes were derived from the interviews with the MHPs. Two MHPs submitted naïve sketches (one was a written naïve sketch, and the other was a drawing) for analysis. The naïve sketches were voluntary and not requested from all participants; key themes were integrated into the results to complement the interview data. Table 2 provides a summary of the themes and sub-themes derived from MHPs, followed by a discussion of the findings. The developed themes are supported by direct quotes from the participants.

**TABLE 2:** Summary of the derived themes and sub-themes.

Themes	Sub-themes
1. Mental healthcare services are viewed as a source of holistic support.	1.1. Promote mental wellness, a nurturing environment and mental health advocacy for students.
2. Mental healthcare provision is experienced as fulfilling and challenging.	2.1. Ensure collective support between colleagues and a reliance on own support. 2.2. Service demands and high expectations from students impact the quality of the services being provided.
3. Mental healthcare services should be student-centred and engaging.	3.1. Well-resourced mental healthcare services should be offered in scenic and tranquil environments.
4. Services should be available, accessible and responsive to address students’ needs.	4.1. A multidisciplinary team approach and the use of various interventions are required when providing mental healthcare services. 4.2. Improved access to the services and students’ mental well-being is needed.
5. Mental healthcare services should integrate monitoring and evaluation strategies.	5.1. Students should provide feedback about the services they receive. 5.2. Mental health practitioners should engage in individual student tracking systems.

*Source*: Adapted from Simangele LL. Strategies to facilitate the mental health of students in a higher education institution [homepage on the Internet]. University of Johannesburg; 2025 [cited 2025 Nov 13]. Available from: https://ujcontent.uj.ac.za/esploro/outputs/doctoral/Strategies-to-facilitate-the-mental-health/9956306507691#file-0^22^

### Theme 1: Mental healthcare services are viewed as a source of holistic support

This theme represents participants’ views and understanding of what mental healthcare services are and what their roles encompass. The sub-theme derived from this theme is discussed below, with direct quotations from participants in support.

#### Sub-theme 1.1: Promote mental wellness, a nurturing environment and mental health advocacy for students

This sub-theme included mental health education, training and awareness programmes being offered by MHPs to students in HEIs. A nurturing environment was also crucial for providing mental health support, guidance and mentorship. Other duties related to advocacy included guardianship, advisership and custodianship, among others. The direct quotes below support the findings:

‘Providing support, through psychoeducation, psychotherapy. And um, medication if necessary for individuals who are experiencing difficulties.’ (MHP10, 25-year-old female)

‘So, with advocacy work it’s taking very specific social or social and psychological problems targeting you know campaigns and information specifically directed to either providing information or letting students know about support and resources.’ (MHP3, 45-year-old female)

### Theme 2: Mental healthcare provision is experienced as fulfilling and challenging

Fulfilment was derived from the rewarding feelings that accompanied the observations of positive changes in students. The challenging experiences were mainly due to a lack of resources. The discussion that follows focuses on the two sub-themes derived from this theme.

#### Sub-theme 2.1: Ensure collective support between colleagues and reliance on one’s own support

Mental health practitioners relied strongly on support from their colleagues and their own resilience. This implied that MHPs in the HEI setting lacked adequate managerial support and care. They purported to employ the same coping mechanisms and therapeutic approaches they teach students. Some individuals engaged in extensive self-reflection, while others sought help from family members, relatives or other professionals; however, all of these were external interventions. Participants stated:

‘I know we definitely recognised we supported with regards to being in student affairs, et cetera, and for example, the Dean is fighting for what we need.’ (MHP6, 59-year-old female)

‘I do my own mindfulness practice. I have physical activity which is important to kind of distress. So, I take care of myself in those kinds of ways.’ (MHP6, 59-year-old female)

#### Sub-theme 2.2: Service demands and high expectations from students impact the quality of the services being provided

Students’ unrealistic expectations were challenging for MHPs, who appeared to be struggling with them. These unrealistic expectations included students making urgent demands to be seen immediately, regardless of appointment systems; instant solutions; expecting MHPs to be available outside of working hours; and assuming that MHPs can intervene in academic decisions. Moreover, the need for professionalism made it difficult for MHPs to raise these challenges with colleagues and management; they feared being perceived as unprofessional and unable to cope with their work. Additionally, MHPs were under pressure to meet students’ unrealistic, urgent demands, resulting in feelings of inadequacy, self-doubt and overwhelm. The following claims offer evidence:

‘For me the negatives come in when students are having very unrealistic expectations.’ (MHP6, 59-year-old female)

‘The pressure to fix things where students sometimes their expectations are not realistic, or they expect you to sort everything out.’ (MHP4, 54-year-old female)

### Theme 3: Mental healthcare services should be student-centred and engaging

Student-centred services refer to services that are tailored to address and meet students’ specific challenges. Students should ultimately be empowered to support one another and make informed decisions about their treatment plans, including what to treat, who to treat, how to treat and when to treat. The passage below provides support.

#### Sub-theme 3.1: Well-resourced mental healthcare services should be offered in scenic and tranquil environments

Appropriate resource allocation includes providing shared workspace within the same building, which facilitates collaboration among MHPs and enables easier student referrals between MHPs. The current lack of space results in insufficient group work, deprived privacy and confidentiality, and overcrowding, all of which negatively impact students seeking help. The following quotes elaborate on this finding:

‘It would firstly be in a very modern looking space, hmm right. A space that that offers a lot lighter and you know just the, the colour scheme and whatever.’ (MHP3, 45-year-old female)

‘One huge building and then of course maybe there will be key people who will be taking intake or whoever the student.’ (MHP12, 36-year-old female)

**FIGURE 1:**
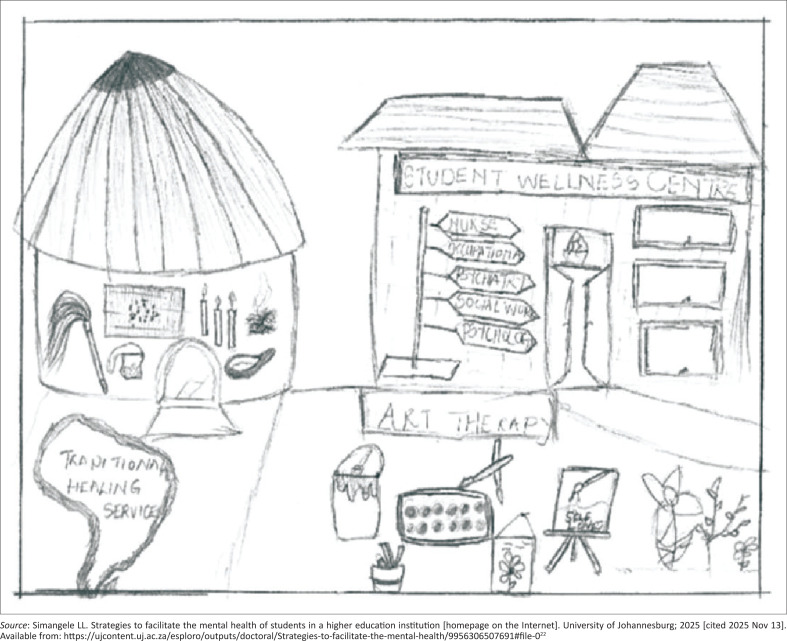
Naïve sketch by mental health practitioner 7, a 27-year-old male.

The following participant’s naïve sketch (Figure 1) elaborates on this finding.

### Theme 4: Services should be available, accessible and responsive to address students’ needs

Participants clarified that for services to be considered available and accessible, two things need to be improved: hiring more MHPs and developing marketing strategies that support mental health. They explained that service responsiveness could be improved by appointing additional specialised MHPs to expand capacity for conducting evaluations, providing crisis intervention, offering individual therapy and facilitating group work. The two sub-themes, along with direct participant quotes, are presented below.

#### Sub-theme 4.1: A multidisciplinary team approach and the use of various interventions are required when providing mental healthcare services

A multidisciplinary team comprises health professionals, including psychiatric nurses, psychiatric doctors, psychologists, social workers, occupational therapists, physicians and pharmacists, who work together to care for students. The use of different interventions, such as peer-to-peer, blended and other approaches, would help increase access to various specialities and reduce external referrals. The following quotations support the findings:

‘On psychological first aid, yeah, because most of the time they are the ones coming into contact first with the student.’ (MHP10, 25-year-old female)

‘A combination of face-to-face on-site kind of access in terms of how students can easily use technology to log in and have a programme that automatically sets an appointment.’ (MHP3, 45-year-old female)

#### Sub-theme 4.2: Improved access to the services and students’ mental well-being is needed

Mental health practitioners suggested that an indication that the services are fulfilling the students’ mental health needs is when they begin to report changes in their coping skills, such as when they become less triggered and managed crises better. This view is supported by the following statements:

‘Obviously, when the population becomes more conscious, the need arises. some form of mental health awareness.’ (MHP2, 33-year-old female)

‘Seeing the impact, the positive impact of it in their academics in coping with the stress that university comes with.’ (MHP10, 25-year-old female)

### Theme 5: Mental healthcare services should integrate monitoring and evaluation strategies

Mental health practitioners emphasised the significance of setting up monitoring and evaluation procedures as a way of assessing the services and providing a voice to the service users. A discussion of the two sub-themes, along with direct quotations to support them, follows.

#### Sub-theme 5.1: Students should provide feedback about the services they receive

All MHPs reported the importance of receiving feedback from students. It was important for them to hear students’ views and suggestions about these services, their work and future planning for mental healthcare services. Participants explained:

‘I think asking for regular feedback is important.’ (MHP11, 38-year-old non-binary)

‘Getting feedback, there should always be a way of getting feedback from clientele, from people that have accessed the service.’ (MHP5, 44-year-old male)

#### Sub-theme 5.2: Mental health practitioners should engage in individual student tracking systems

The importance of student tracking, to understand who uses these services, for what they are consulting and what the outcome is post-therapy, was emphasised. Feedback serves as an indication of what is working that MHPs can continue using, and what is working but needs to be improved. Participants shared:

‘Checking if Ummm the, the majority of the students who are using the services and also are new to the service Ummm of how they feel about it.’ (MHP8, 26-year-old female)

‘I think that’s the most important is to check from the mental health what they are kind of experiencing, what is coming through, what isn’t working, what is working and have to change accordingly.’ (MHP9, 25-year-old female)

## Discussion

This study was conducted to explore the experiences of MHPs when providing mental healthcare services to students of HEIs. Despite the critical role they play in supporting students with mental health challenges, limited research has examined their experiences.^25^ The findings of this study contribute to addressing this gap by highlighting the multifaceted nature of MHPs’ roles and the challenges they encounter that negatively influence service delivery. These core macro skills, fundamental to mental health care practice, require kindness, caring attitudes and empathy. When it comes to the physical setting where these services are offered, there is a need for modern, attractive buildings to make students feel welcome. Mental health practitioners supported this, but financial constraints prevented this from happening. Studies have reported that offering these services in settings with modern buildings with sufficient space has improved access to mental healthcare services.^26,27^ The increase in challenges related to the mental health of students makes it mandatory for mental healthcare services to be established in HEIs.^28^ A Canadian study showed that access to adequate mental healthcare services is a need for youths who enrol in post-secondary education.^29^ Thus, HEIs rely on mental healthcare services for assistance with treating and managing students presenting with mental health challenges.^4,7^ Also, the effects of the COVID-19 pandemic on students’ psychological well-being worldwide were notable, making these services a demand for HEIs.^30^ Research has shown that mental health counselling services in universities are important and serve multiple purposes, including assisting students to cope and improve their academic outcomes.^31,32,33^ The experience of MHPs in providing mental healthcare services was both good and challenging. The positive was linked to student academic success, while the challenges arose from the attitudes and expectations, parents’ roles and responsibilities, their own mental health issues and resource scarcity.^31,34^ The parents’ role includes their involvement in supporting their children’s education, the responsibilities they assume in managing academic and personal matters, and any mental health issues they themselves may experience, which can influence the support they provide. As part of coping with the challenges of the work they do, MHPs verbalised relying on each other for support and their families. Research showed similar findings, that cultivating self-care fostered an increased sense of general wellness and mitigated the mental challenges experienced by MHPs.^34,35^ Also, positive self-care practices such as socialising with others, including taking time away from work, practising healthy sleep habits and incorporating healthy diets^36^ were recommended. Mental health practitioners emphasised the importance of mental health awareness among students and staff members. Therefore, information sharing about services was supported to empower service users with information on where, when and how to access these services, thereby addressing concerns about late access.^37,38,39^ However, poor resource allocation and availability continue to impact these services, and according to research, 71.5% of students did not have their mental health needs met through counselling.^32,40^ Mental health practitioners suggested collaboration and working closely with all stakeholders to improve service access. Research supported the provision of holistic services through a multidisciplinary approach and the active engagement of students with these services.^26,41^ Also, financial support was emphasised to fund and establish mental healthcare services in HEIs. These services were reported to be expanding at a slower rate than the increase in students’ mental health demands.^42,43^ Thus, the expansion of resources, both financial and employment of more MHPs, was supported.^26,31^ Lastly, evaluation and feedback from students were emphasised by MHPs, and research showed that having students’ and stakeholders’ buy-in and input about these services was important for the evaluation.^38^ Establishing services that students can identify with will help encourage ownership and a sense of belonging.^27^ Therefore, increasing active engagement will help to capture the voices and experiences of the students to improve mental healthcare services.^4,30,31,44^

### Implications for practice

Mental health practitioners reported that these services were offered to support students battling with mental health challenges. This support took various forms, which meant more responsibilities for MHPs.^31,32^ Therefore, offering these services helps to identify, prevent and treat mental health challenges effectively.^33^ Organisational resources, support and caring for everyone were important.^36^ Also, providing employees with support and establishing employee wellness services were raised. A lack of such resources negatively impacted the functionality of MHPs, as they require sufficient support to perform their duties.^31,45^ The issue of resource scarcity and financial constraint resulted in MHPs’ failing to meet the mental health needs of students of HEIs. Therefore, to mitigate these challenges, MHPs recommended capacity building through offering courses on mental health to both staff and students. These courses included training in mental health first aid, mental health emergency screening and triaging for prompt responses to all mental health emergencies and crises, and peer-to-peer support.^43,46^ Studies suggested interventions like referring students to external service providers for long-term treatment.^32^ Mental health practitioners supported this due to poor staff capacity, which was affecting the provision of long-term therapy to students, and they suggested liaising with and coordinating these external referrals to ensure easy access for students. Buddy systems (pairing students) to help students cope and connect^32^ and mentor–mentee programmes for student support^31,47^ were supported to reduce the workload for MHPs while ensuring continuous mental help. Improving mental health literacy through mental health training, focusing on basic symptoms and a 24-h helpline, was supported to boost the students’ confidence in seeking help and to reduce mental health stigma.^43^ Academic commitments hindered students’ access to help^32^; therefore, the use of technology was further supported.^48^ Similarly, other findings indicated that digital interventions offer a more appealing option of support for students, including online counselling, mental health apps, virtual support groups, psychoeducational resources and teletherapy sessions, which provide accessible and flexible avenues for addressing mental health concerns;^49^ students agreed that digital interventions were effective.^32,40^ The findings indicate that MHPs relied heavily on support from colleagues and their own resilience to cope with workplace challenges. This reliance highlights a gap in organisational support, suggesting that managerial support and institutional care for MHPs in the higher education setting are currently insufficient. It is therefore recommended that HEIs strengthen managerial structures, policies and support systems to better address MHPs’ professional and emotional needs. Lastly, active student participation in the development and implementation of mental healthcare services was recommended to ensure the rendering of effective and efficient mental healthcare services.^3.13^ This was further supported by literature.^38,50,51^ Therefore, HEIs must fund mental healthcare services to ensure adequate resource allocation. When MHPs are well supported to do their job, students will receive the help needed to cope and succeed in completing their studies.^13^

### Strengths and limitations

A strength of this study was the inclusion of all MHPs, ranging from nurses, psychologists and social workers working at an HEI. The limitation of the study is that the research was conducted within a single HEI, which may limit the transferability of the findings to other institutional contexts.

## Conclusion

This study revealed the challenges faced by MHPs and how those resulted in their own mental health being affected. Resource scarcity continues to be a barrier to providing quality care to students of HEIs affected by mental health challenges. Higher education institutions must do more to adequately support MHPs and enable them to render these services effectively to students.
